# The Medical Epitome Series

**Published:** 1902-12

**Authors:** 


THE MEDICAL EPITOME SERIES. Genitourinary and Venereal Diseases. A Manual for Students and Practitioners. By LOUIS E. SCHMIDT, M. Sc., M. D., Associate Professor of Genitourinary Diseases, Chicago Polyclinic. Series edited by V. C. Pedersen, A. M., M. D. Recently assistant demonstrator of Anatomy, College of Physicians and Surgeons, Columbia University, New York. Duodecimo, pp. 249. Illustrated with 21 engravings. Lea Brothers & Co.: Philadelphia and New York. 1902. [Price, cloth, $1.00 net.]

This little volume which helps to make up what is known as "The medical epitomé series" is no more or less than a mine of information upon the subjects with which it deals. In these busy days the older practitioners, and for that matter the younger ones, and even the specialists, find that at times it is necessary to look up subjects on which from not very close acquaintance they are a little rusty, if not considerably foggy.

In such a condition the frame work being up, what is wanted is the filling and this should be handed to us in the most concise, up-to-date and abbreviated manner. Such a field this series is expected to occupy. In this number the subjects are well dealt with and in a way that shows the author to have an extensive experience. He attained good results and has published them for the benefit of others. The work is well gotten up, convenient to carry in the pocket and should be found in the library of every practitioner of medicine. J. H. D.

